# Effect of Ear Infections on Hearing Ability: A Narrative Review on the Complications of Otitis Media

**DOI:** 10.7759/cureus.27400

**Published:** 2022-07-28

**Authors:** Abdullah Jamal, Abdulla Alsabea, Mohammad Tarakmeh

**Affiliations:** 1 Otolaryngology, Zain Hospital, Kuwait City, KWT; 2 Cardiology, Mohammed Bin Khalifa Bin Salman Al Khalifa Specialist Cardiac Centre, Awali, BHR; 3 Otolaryngology, Jaber Hospital, Kuwait City, KWT

**Keywords:** hearing loss, hearing ability, children, acute otitis media, ear infection

## Abstract

Otitis media (OM) is an infection that occurs in the middle ear and can affect the structures around the ear, as well as the auditory system. It is one of the most frequent diseases affecting young children each year, especially those aged around six years, due to the anatomical structure and developing immune system.

Although some cases of OM resolve spontaneously, children often need medical care since difficulties persist with such infections. The incidence of OM is higher among children than adults, and therefore, their speaking, hearing, and learning capabilities and general development are impaired by recurring middle ear infections.

The literature over the last 40 years has documented the impact of early auditory deprivation produced by early OM with effusion (OME) on central auditory processing (CAP). This study aimed to review the impact of acute ear infections on hearing capacity, assess the complications of OM, and document the scientific evidence around the implications of early OME-induced hearing loss in children.

Studies have reported the association between hearing loss owing to early OME and alterations in CAP in both children and adolescents. The auditory foundation enables hearing capacity, but this is continually depleted. Therefore, the use of strong antibiotics, sound amplification, hearing rehabilitation, and ear surgery in children must be improved.

## Introduction and background

A middle ear infection is referred to as acute otitis media (AOM). Otitis media with effusion (OME), chronic suppurative otitis media (CSOM), and AOM all fall under this spectrum of illnesses. After upper respiratory infections, AOM is the second-most frequent paediatric diagnosis in the emergency room. Otitis media (OM) can strike at any age, however, it is most frequently diagnosed in infants between the ages of six and 24 months [1].

The incidence of AOM, which is typically self-limited, is 10.9%. Chronic OM (COM), which has an incidence of 4.8%, [2-4] is more challenging to identify and treat. Rates of complications can range from 5% to 12.5% and might be extracranial or intracranial. The most frequent extracranial side effects of OM include mastoiditis, labyrinthitis, subperiosteal abscess, facial paralysis, and labyrinthitis. Meningitis, lateral sinus thrombosis (LST), cerebral abscess, otitic hydrocephalus, extradural abscess, and encephalitis are the most frequent intracranial complications (ICCs) of OM [5-7].

Consequently, this study aimed to consider the effects of ear infections on hearing abilities and the repercussions of OM.

## Review

Ear infections and OM

Medical problems due to viral or bacterial infections in the middle ear, like acute nonsuppurative OM and acute suppurative OM, are known as ear infections. OM is frequently caused by other disorders, such as allergies, colds, or influenza, which cause congestion in the auditory, nasal, and throat passages. Infection can arise in the middle ear, which is a tiny space within the ear below the eardrum that divides the middle ear from the outer ear (eardrum) when bacteria from the nose and throat enter. Ear pain (that can vary in severity and tolerability), discomfort and nervousness, malaise, sleep disturbances, discharge of a yellow sticky liquid from the ears when an ear infection is caused by tympanic membrane rupture, higher body temperature, hearing problems, and a feeling of pressure in the ears are all common symptoms of OM. Otitis is a general term for ear inflammation or infection, which encompasses both human and animal ear diseases. Infections are classified into three categories: otitis externa, OM, and otitis interna [8].

Otitis externa, sometimes known as “swimmer’s ear,” is an inflammatory condition that affects the outer ear and ear canal. Ear tugging or touching is painful when this ailment is present.

OM, as defined earlier, is an inflammation of the middle ear. The ear is infected (e.g., with bacteria) or obstructed in OM, which affects the middle ear, due to fluid accumulation behind the space behind the eardrum, which is normally filled with air and sterile. This damage most commonly affects youngsters, and it may necessitate surgical intervention in the form of eardrum surgery, which involves inserting a tube into the eardrum to reach the required location.

A sudden onset of this disease is referred to as AOM. AOM occurs when pus and mucus deposit behind the eardrum, obstructing the eustachian tube, and is caused by colds, allergies, or bacteria/viruses. This may cause ear pain and fever. The disease is referred to as OME when the fluid remains in the middle ear for weeks after the ear infection has been treated. OME often occurs after AOM, but it also may occur as a result of eustachian tube dysfunction in young children in the absence of a preceding AOM. The fluid may remain in the ear for several weeks to months. Persistent ear infections, if left untreated, can have catastrophic implications, including temporary hearing loss [9].

The inner ear contains sensory organs that are important for hearing and balance. When the inner ear becomes infected, it can cause dizziness, which is a common symptom [10].

Infections of the middle ear (especially OM) are common in children. By the age of three, it is believed that at least half of all children have had at least one bout of ear infection [11]. Because it is a frequent ailment in children, parents and practitioners must monitor indications and symptoms [12]. This is typically accompanied by other upper or lower respiratory tract disorders [13].

One of the most common side effects of OM is conductive hearing loss [14]. Hearing loss can result in speech and language problems, as well as academic delays [15]. Hearing impairment can lead to behavioral issues which can also elevate the risk of anxiety and depression [16]

OM adversely affects children’s quality of life, and depending on illness severity, it may also have a greater influence on medical care and service providers [17].

Complications of OM

OM occurs when the tube that links the ear to the neck swells for various reasons, including the common cold, causing fluids to become trapped inside the ear (providing a good environment for the reproduction of ear infection-causing germs). Because newborns and young children’s ears are tiny and prone to obstruction, this facilitates OM. While most ear infections do not have long-term consequences, repeated ear infections can result in the following catastrophic consequences [18]:

1-Mild hearing loss: Mild hearing loss that comes and goes is a typical co-occurring disease with ear infections; however, hearing normally recovers after the infection is cleared. Hearing loss can be worsened by ear infections or fluid accumulation in the middle ear. Permanent hearing loss can be caused by permanent injury to the eardrum or other components of the middle ear.

2-Delay in social skill or speech development: Infants and young children with temporary or permanent hearing loss may experience delays in social skills or speech development.

3-Infection spread: If an infection is left untreated or does not respond to therapy, it can spread to neighboring tissues. Mastoiditis (infection of the mastoid bone, which protrudes behind the ear) can cause bone deterioration or pus-filled cysts to grow. A serious middle ear infection can spread to other tissues in the skull, such as the brain or membranes surrounding the brain, in rare circumstances (meningitis).

4-Eardrum rupture: Most eardrum ruptures heal within 72 hours, while surgery may be necessary in some situations.

5-Inflammation and disintegration of the mastoid: Inflammation and disintegration of the mastoid (airy bone cells behind the ear) are further issues that can be medically treated with antibiotics or surgically. The inflammation can extend to the inner ear, causing dizziness, ringing in the ears, hearing loss that is nearly irreversible, and even meningitis (inflammation of brain membranes, which results in neck pain with stiffness, inflammation, high temperature, severe headache, brain abscess, and paralysis of the seventh facial nerve that passes into the middle ear) [19].

6-Hearing and facilitating body balance are principal functions of the human ear. The outer ear receives sound through the pinna; the middle ear transfers sound from the outer ear as vibrations to the interior; and the inner ear conveys sound vibrations from the middle ear to the brain via nerves [9].

7-The body secretes a mucous fluid that collects behind the eardrum in OM owing to bacterial or viral infections, causing increased pressure on the middle ear and drum bulging, which produces discomfort. Infections can also be caused by dysfunction of the eustachian tube, which links the middle ear to the throat and balances pressure between the outer and middle ear. The existence of a defect in this tube hinders its normal functioning, inhibiting normal fluid outflow from the middle ear and causing fluid collection behind the eardrum. Bacteria and viruses can proliferate in the ear, producing inflammation [8].

OM is one of the most frequent childhood illnesses. Because children are more susceptible to OM than adults due to severe cold and weaker immune systems; OM causes significant discomfort in young children, which worsens as they sleep. When the pressure in the middle ear increases and treatment is delayed, problems such as eardrum rupture or hearing loss can occur.

Antibiotics must be given to children with a severe bacterial infection (i.e., when the fluid behind the eardrum is no longer clear). Scarring in the middle ear develops when the infection recurs, reducing hearing capacity. When a child’s hearing is impaired and fluid lingers behind the eardrum, a tiny incision is made in the eardrum and a small ear tube is inserted to clear the fluid [20].

The collected water in the form of mucus is drained since its presence for an extended length of time may induce chronic OM, and a tube is inserted into the ear to ventilate it. Due to their incapacity to pinpoint discomfort, newborns have a tough time detecting ear infections. As a result, sleep difficulties, appetite loss, and behaviors, such as pulling ears or refusing a bottle of milk because the pressure on their ears makes swallowing difficult, must all be monitored. Most ear infections resolve in three days, however, symptoms may continue for up to a week [19].

OM is not very different between adults and children. When fluid congestion and buildup develop in the eustachian tube because of allergies or colds, inflammation ensues. Whereas the eustachian tube should typically solely contain air, bacteria and viruses proliferate and spread to the middle ear because of fluid buildup and pressure on the eardrum. When an infection is left untreated, intense pressure builds up, resulting in a hole in the eardrum, pus leakage, and suppurations that spread to other body parts. In certain circumstances, this hole may close; if it does not, microbes and water from the outside may enter the ear. Disease symptoms in adults vary depending on whether the infection is viral or bacterial or in the throat or nose; ear pain occurs, with a hearing problem or a sound-like ringing in the ear. Fever appears, with headache and dizziness; nausea, upset stomach, and vomiting may occur, and in the case of infection, the eardrum may rupture [21].

Recurring ear infections can lead to major problems such as hearing loss. This is common with middle ear infections and mild hearing loss but disappears once the infection heals; repeated ear infections cause severe hearing loss due to the presence of fluid in the middle ear, which can lead to permanent hearing loss if the middle ear drum or other parts of the middle ear are damaged.

Hearing loss, whether temporary or permanent, affects a child’s capacity to communicate and understand their surroundings. Non-responsiveness to therapy can cause viral infection to spread to the projecting bone behind the ear (so-called “mastoiditis”). The infection may spread to the brain or nearby tissues, causing bone damage or pus production. Eardrum rupture is a possible side effect; however, the infection normally clears in a few days. Surgical intervention may be necessary for rare circumstances [8].

In summary, middle ear infections usually occur during or after upper viral respiratory infections, particularly the common cold. Inflammation and swelling at the back of the throat, particularly the eustachian tube, are symptoms of the condition. The eustachian tube swells and is unable to equalize the pressure in the middle ear space. Because of the pressure buildup within a tiny region of the middle ear, regular secretions are unable to be flushed out as they typically would. Pain, dizziness, and temporary hearing loss can be caused by negative pressure and excess fluid buildup [22].

Middle ear infections can occur in anyone with a cold. Middle ear infections are more common in younger children for two reasons. Their immune systems are less developed, making it more difficult for them to combat respiratory infections, and their eustachian tube is more horizontal, making it more difficult to empty fluid. Chronic infection of the adenoids or tonsils is another risk factor for middle ear infection. Viral or bacterial invaders can readily travel to the middle ear since these glands are near the eustachian tube. An otolaryngologist may recommend surgical removal of these glands (tonsillectomy or adenoidectomy) to prevent infections from spreading in the future. Middle ear infections are likely to improve once the surgical region has healed and the middle ear space has been aired [20].

The pneumatic otoscope, which allows viewing of the ear, can be used by the physician to assess the condition of the outer ear and eardrum. A puff of air in the ear can also be used to detect problems, and the movement of the drum can be studied. When an infection recurs, the physician performs a hearing test to check whether there is hearing impairment [21].

Physicians recommend cleaning the ear with care and gentleness, especially while using cotton buds to clear wax. Sharp equipment should not be used for cleaning the ear, and the ears should be thoroughly dried after each shower, since water left in the ears may encourage bacteria and other microbes to enter. Personal hygiene is essential for maintaining health and preventing colds and flu. In general, a physician should be consulted if high temperature is accompanied by shivering or discomfort in the ear that lasts for many days without improvement, especially if there is a tumor or fluid discharge or if hearing impairment or loss, severe sore throat, or dizziness is present. Treatment (antibiotics, pain killers, or ear drops) is recommended based on the patient's condition, and chronic infections, as well as hearing issues owing to permanent weakening or complete loss, may need the implantation of tiny tubes in the eardrum [23].

Hearing ability

Auditory experience is critical for the development of the central nervous system. Although sensory functions can be determined naturally, hearing tests require training [10].

OME is one of the most prevalent pediatric disorders [24]. It is described as an inflammation of the middle ear accompanied by excess fluid in the middle ear with no signs or symptoms of the middle ear accompanied by excess fluid in the middle ear with no signs or symptoms of acute infection, such as ear discomfort, fever, or tympanitis, and can result in mild, permanent, or fluctuating conductive hearing loss [25]. Hearing loss can range from 10 to 40 dB and is commonly felt as a widening of the air-bone gap. Approximately 15% of adolescents with OM have hearing thresholds >25 dB degree of hearing loss [21]. The auditory system is fully functional before birth and remains so for the first two years of life. Changes in the physiology and functional development of the hearing organ may occur if there is a reduction in auditory input through peripheral structures during this time [26]. Recent research has found that core hearing abnormalities, such as defects in temporal processing, binaural hearing, and binaural speech perception, can remain as noise long after OME has disappeared [27]. By measuring absolute and interpeak latencies, the auditory brainstem response (ABR) provides information on the integrity of the auditory brainstem. Inter-peak latencies (I-III, III-V, and I-V) are widely regarded as measurements of central nervous system conduction time [28].

Animal research into conductive hearing loss has demonstrated that OM events can cause major alterations in the structure and function of the central auditory nervous system (CANS). Tucci et al. investigated the effect of sonic exposure during the 2-deoxyglucose (2-DG) integration phase on 2-DG uptake levels in CANS in jerboas with unilateral conductive hearing loss caused by the removal of the malleus [29]. They discovered that 2-DG absorption was reduced in the ipsilateral cochlear nucleus and contralateral inferior knob, as well as in the nuclei of the upper olive complex on both sides, confirming the link between conductive hearing and reduced CANS neuron activity. Previous ABR studies had focused on children with early bouts of OM (18 months) who did not require invasive tube implantation and found indications of brainstem involvement in the form of extended ABR waves, following middle ear disease therapy [10].

The long-latency auditory evoked potential (LLAEP) exhibits the activities of discrimination, attention, and integration by reflecting the neuronal electrical activity of sites in the thalamus and auditory cortex. P300 is an endogenous LLAEP that consists of a positive wave with a post-stimulus delay of around 300 ms, suggesting activity in brain areas that control certain activities such as attention and memory [30]. Long-term impacts of hearing deprivation may include a reduced degree of functioning across the auditory system. On the P300 scale, there is evidence that children with voice disruptions and attention problems suffer delays.

Despite this, few studies have measured P300 in children with OM to investigate the consequences of early-onset middle ear infection on the cortical level in these individuals [10].

Effects of ear infections on hearing ability

OM is a fairly widespread ailment that affects approximately 11% of the world’s population (about 700 million people) on an annual basis [31]. Children make up the bulk of those infected, accounting for over half of the cases [32]. In all, 31 million people with AOM develop CSOM each year, including >7 million children [29]. Hearing loss affects more than half of individuals with CSOM [20] or around 0.3% of the world’s population [20,33]. Given the high rate of conversion of AOM to CSOM, these findings emphasize the necessity of early detection and treatment of the auditory system which consists of several systems that enable an individual to receive and evaluate sounds, including sensory organs, nervous system auditory pathways, and brain regions responsible for hearing [10]. The CANS and the brain assess the internal response to sound stimuli, and a reaction is generated, while the peripheral auditory system receives and analyzes sound waves released by vibrations from the surroundings [34,35]. Auditory processing is described by the American Speech-Language-Hearing Association as the efficiency and efficacy with which the central nervous system utilizes auditory information. Auditory processing disorder, on the other hand, is defined as difficulty in the central nervous system’s perceptual processing of auditory information and neurobiological activity that results in the appearance of auditory electrophysiological potentials that are unrelated to changes in cognitive speech or other associated factors [36]. A prior study found a link between OM experiences in childhood and auditory sensory alterations, as well as an elevated risk of future speech difficulties in children who had OM events [37]. Infection (bacterial or viral), eustachian tube dysfunction, decreased immunological system, upper respiratory infections, environmental difficulties, and, in certain cases, social problems are all variables that contribute to OM [38].

Inflammation is most prevalent in childhood and adolescence and gradually fades with maturity. Between the ages of one and five years, about two-thirds of children have had at least one episode of secretory OM (SOM). Because of eustachian tube flattening, this disease is quite frequent in young children, resulting in recurring bouts of OM [39]. If medical assistance is not sought early, OM can result in hearing loss because of fluid collection in the middle ear, which makes sound transmission across the bone more difficult due to sound energy dissipation. Consequently, the child suffers from mild to severe conductive hearing loss, which is sometimes referred to as a fickle personality [40]. Although there are moments of normal hearing in OM, the fluctuating nature of these intervals causes inconsistent acoustic stimulation of the CANS, resulting in distorted sound perception and speech discrimination, especially in noisy surroundings. Additionally, voice awareness abilities may be harmed, significantly impacting school achievement [41].

Numerous studies have shown that children with and without OM have substantially different voice problems. Generally, children with OM perform worse in cognitive and auditory processing tests than children without OM, and they have a higher rate of scholastic issues, owing to alterations in reading and writing [42]. The criteria for elective ear, nose, and throat (ENT) surgery has been changed to take into account the effects of childhood OM, particularly in terms of speech acquisition and development. Myotomy, or the installation of a ventilation tube, has become the most common operation in young children. In the United States, almost 500,000 children received surgery in 1994. This procedure removes fluid from the middle ear and restores hearing [43].

However, a single surgery for SOM is insufficient to achieve appropriate CANS stimulation. In addition to genetic variables, cognitive and linguistic development is influenced by social (e.g., relationships between children and their parents, family, and school) and behavioral factors (e.g., reading, listening to music, visiting theatres and parks) [21]. Due to a lack of educational materials, proper role models, and parental aid with sensory, verbal, and literacy development, children from low-income homes have inferior cognitive development and school performance than children from high-income families, according to several studies [44].

Curi and Menezes-Filho noted that school choice is influenced by the family’s economic status and educational level, as assessed by the public’s capacity to send their children to the school of their choice. There is a link between parental education and school choice, according to the researchers [44]. In Brazil, 55% of private school parents have finished at least secondary education, compared with <20% of public school parents. As a result, private school students have a better socioeconomic standing than public school students. The family’s socioeconomic and educational standing are elements that might lead to the child’s overall development being delayed in this situation. These effects can be amplified, particularly in children who have had several bouts of sensory hearing loss owing to middle ear effusions [45].

SOM is a clinical condition defined by middle ear effusion without eardrum perforation and a three-month-long acute infectious phase. It affects children between the ages of three and nine years.

The most common sign is hearing loss, which is frequently detected by parents or instructors as a lack of attention and interest, repeated requests for the message, and poor academic achievement. The pathogenesis is complex, with eustachian tube dysfunction and upper airway infections of allergic, viral, or infectious origin having the highest prevalence. The immune system matures and the auditory tube strengthens with age, lowering the disease incidence [46].

Otoscopy was used for diagnosis, which was then confirmed by audiological assessment. Otoscopy can reveal eardrum retraction, which is characterized by limited mobility, opaque appearance, and aberrant color. Mild-to-severe conductive hearing loss, generally bilateral, with a type B tympanic curve is diagnosed on auditory assessment. Hearing loss is sporadic, transient, and asymmetric [19].

Depending on the health of the middle ear and the patient’s medical history, treatment may be clinical or surgical. The most frequent therapy for a middle ear infection is tympanostomy, which involves inserting a tube into the middle ear to drain fluid and restore hearing [21].

Even years after the OM has cleared and pure-tone thresholds have recovered to normal, children with SOM may show deficiencies in binaural hearing and auditory ability [24].

The central auditory processing (CAP) battery assesses how well the central nervous system can process changing sound stimuli [46]. To measure auditory information processing, gaining additional data on CANS functions, developing a more accurate diagnostic method, and identifying diagnosis and treatments, both behavioral and electrophysiological techniques, are now advised [47]. Auditory abilities and generated auditory potentials, such as auditory brainstem click response, post-frequency response, and delayed auditory skills, may all be assessed via CAP behavioral testing (N1-P2-N2 complex and P300). These tests extract signals that directly indicate brain activity in the auditory pathway, from the auditory nerve to the cortex in response to an auditory stimuli providing further information regarding the nerve to the cortex, in response to an auditory stimuli, providing further information regarding CANS functioning [48].

Since children are deprived of appropriate auditory input early in life, their CANS may shift, and their perceptual sensitivity to auditory information processing may be diminished later in life.

Recent research in people and animals has found that sensory deprivation during development causes long-term cellular abnormalities in the auditory cortex as well as a decrease in behavioral performance [18].

OME is a middle ear inflammation defined by middle ear fluid and the absence of signs or symptoms of acute infection or tympanic membrane rupture. The tympanic membrane’s mobility is reduced, and sound waves are transmitted more slowly [49]. Because OME is the most prevalent reason for medical consultations and procedures in children, its high incidence in early life has sparked discussion regarding its influence on the development of the human auditory system. It is called “early” when it affects children in their first five years of life [50]. Hearing development is assumed to be impaired by temporary and variable hearing loss produced by the illness at this period of life [37], and it is considered a kind of hearing deprivation [51]. The first study to show a link between early auditory deprivation and abnormalities in long-term auditory development was published in 1962. The consequences of hearing deprivation on a patient’s delayed behavior are described in this report.

Several studies [21] have sought to elucidate the long-term effect of early OME-induced hearing loss on CAP since that time. Despite the enormous number of research published over the last 40 years, some disputes persist [52]. Several scholars have questioned these debates, claiming that the bulk of the research was retrospective in nature, making it difficult to establish a precise link between the disease and the result [53]. As a result, longitudinal investigations are needed to see if the negative effects of OME remain after tonal thresholds have been restored [54].

The wider perspective AOM is uncommon in European and North American children older than seven years. It is more likely to occur within the first year of life and becomes less prevalent in succeeding years.

It is a frequent ailment that leads to many doctor appointments and antibiotic prescriptions. Clinically, it is diagnosed based on objective findings on physical examination (otoscopy), as well as the patient’s history and current signs and symptoms. To help in the diagnosis of OM, diagnostic equipment such as a pneumatic otoscope, tympanometry, and acoustic reflectometry are available. When compared with ordinary otoscopy, pneumatic otoscopy is the most trustworthy and has greater sensitivity and specificity, while tympanometry, as well as other modalities, can help with the diagnosis if pneumatic otoscopy is not available.

The burden of AOM varies significantly among countries, as it does with most infectious diseases, with the primary distinctions being the prevalence of suppurative complications such as mastoiditis and meningitis, as well as sequelae such as hearing loss owing to CSOM. CSOM is a leading cause of preventable loss of hearing, especially in developing countries, and a cause for serious concern, especially in children, because it has long-term implications on early communication, auditory processing, language development, educational progress and achievement, and psychosocial and cognitive development.

The World Health Organization (WHO) estimates that 28,000 fatalities per year are caused by OM complications, based on prevalence studies that differ substantially in illness definition, sample methodologies, and methodological quality. Meningitis and brain abscess are the most common causes of mortality in OM and CSOM. Furthermore, the WHO estimates that between 65 and 330 million people have CSOM (display indications of CSOM), with 50% of them having hearing loss.

Hearing loss (both conductive and sensorineural) is the third most common chronic illness in older individuals in developed countries, behind hypertension and arthropathy, with significant physical and mental health consequences. There is a scarcity of data on the adult population of less developed countries.

## Conclusions

By the age of four years, it is estimated that up to 80% of children will have OM. Early onset of OM has been linked to a higher risk of recurrent episodes, which are likely to have long-term consequences. To properly assess the need for treatments targeted at lowering acute OM incidence, and the health, social, and economic impact, a better understanding of the incidence and prevalence of acute OM and its consequences across ages and geographical areas is required.
